# *Clostridioides difficile* Is Not Difficult to Predict in Hospital Settings

**DOI:** 10.1017/ash.2021.76

**Published:** 2021-07-29

**Authors:** Kinta Alexander, Frances Petersen, Sean Brown

## Abstract

**Background:** *Clostridioides difficile* is a gram-positive bacteria that is the most common cause of hospital-associated infectious diarrhea among traditional and nontraditional high-risk populations. Excess healthcare costs associate with *C. diff*icile infection (CDI) prevalence, morbidity, and mortality is shown to economically impact the US healthcare system with at least an additional $1 billion in annual cost. Exposure to antimicrobial agents resulted in increased risk for hospital-onset CDI (HO-CDI) at an inner-city hospital during 2010 and 2011. **Methods:** A retrospective case-control study of all persons with HO-CDI in the MICU was conducted at an inner-city hospital between January 1, 2010, and December 31, 2011. A patient was considered to have developed HO-CDI if diarrhea developed after 72 hours of admission into the MICU and a confirmed laboratory stool specimen for *Clostridioides difficile* infection (CDI) was obtained. A non–HO-CDI person was randomly selected using “risk set sampling.” After the application of inclusion and exclusion criteria, 88 cases were eligible for the study. Of these cases, 29 met the definition for HO-CDI, and 59 met the definition for non–HO-CDI. The relationship between antimicrobial use and the development of HO-CDI in patients in the MICU at an inner-city hospital was investigated using a logistic regression model in which the variable of total antibiotics was used as a possible predictor for predicting a positive HO-CDI. **Results:** Logistic regression was utilized to determine the relationships between selected study variables and presence or absence of HO-CDI. Total antibiotics was significantly related to HO-CDI. The results of this analysis showed that total antibiotics was a significant predictor for HO-CDI. The total value of the coefficient B for this predictor was 0.47, and the exponentiated value (exp[B]) of this coefficient was 1.60 (95% CI, 1.08–2.35). In this sample, patients who had 1 or more antibiotics were at a 60% greater risk of having a positive HO-CDI culture. There was a significant association between the use of metronidazole and HO-CDI (p < .001). **Conclusions:** Antimicrobial stewardship is an integral part of patient safety. The findings from this study were instrumental in the implementation of a fledging antimicrobial stewardship program and the use of evidence-based practices at this inner-city hospital.

**Funding:** No

**Disclosures:** None

